# Medical Society of the County of Erie

**Published:** 1910-02

**Authors:** Franklin C. Gram


### SOCIETY PROCEEDINGS.

### Medical Society of the County of Erie

Reported by FRANKLIN C. GRAM, M. D., Secretary.

THE eighty-eighth annual meeting of the Medical Society of the County of Erie was held in the Young Men's Christian Association auditorium, 85 West Mohawk street, Monday, December 20, 1909, at 8.15 p.m.

The president, Dr. Charles A. Wall, called the meeting to order.

The minutes of the October meeting were approved, as published, without being read.

The regular order of business was then suspended, and the president called for the report of the Committee on Membership.

Dr. T. H. McKee, chairman, presented a list of applicants all of whom were recommended by his committee, and as each name was presented to the society, the secretary, upon vote of the meeting, was instructed to cast the ballot of the society for the candidate, whereupon the president declared each one duly elected.

The names and addresses of the newly-elected members are as follows: Frank L. Watkins, 404 Michigan street; Louis J. Beyer, 972 Michigan street; Loren H. Staples, 121 E. Ferry street; Alexander Allan, 453 Richmond avenue; John J. Drake, 1137 South Side Parkway; R. M. Root, 40 Krettner street; J. Walter Fitzgerald, 274 E. Utica street; Victor A. Pchellas, 1481 E. Genesee street; Nelson W. Bodenbender, 4 W. Parade avenue;

Joseph S. Lehman, Clarence Center; John C. Thompson, 666 Auburn avenue; Benjamin A. Gipple, Main street, Alden; T. H. Johnston, Brant; James J. Brown, 641 S. Park avenue; Nelson G. Russell, 469 Franklin street; Norman Leonard Burnham, 478 Franklin street; Henry Adsit, 412 Ashland avenue; Cyrus S. Siegfried, 6 N. Pearl street; George J. Eckel, 145 Allen street; Guy L. McCutcheon, 1028 Elmwood avenue; James E. Culbert, 125 Seymour street; Charles P. Eller, 1440 Jefferson street; George T. Tyler, 127 Allen street; Charles W. Banta, 358 S. Division street; L. H. Krombein, 217 Masten street; Arthur R. Gibson, 778 E. Genesee street; Frederick M. Boyle, 745 Abbott Road.

The attention of the society was called to the fact that, with the twenty-seven members just elected, the total number of members elected during the year 1909 was ninety-one. This announcement called forth hearty applause as well as a vote of thanks to the membership committee, and especially to its chairman, Dr. T. H. McKee, for the excellent showing. The dues of the newly elected members were ordered to be credited for the year 1910.

The minutes of the council meetings, held November 1, 1909, December 6, 1909, and December 18, 1909, were read by the secretary, and were adopted, together with all the recommendations therein contained, except the question of a permanent meeting place for the society for the year 1910.

The adoption of these minutes carried with it also the acceptance of the resignation of Dr. J. Grafton Jones, dated December 17, 1909, the treasurer having reported that Dr. Jones had paid up to the end of the year.

It also carried with it the adoption of a rule recommended by the council as follows:

"Chapter XIII, Section 2.—Rules and regulations for the government of the society and the administration of its affairs, not repugnant to the Constitution and By-Laws of the Medical Society of the State of New York, and any of these by-laws may be adopted, changed, amended or suspended at any regular meeting by a three-fourths vote, twenty members being present."

The rule was presented later by President Wall.

Dr. John H. Grant, chairman of the Board of Censors read his annual report as follows:

*To the Medical Society of the County of Erie:*

The Board of Censors respectfully submits its annual report for the year 1909:

The Board met January 11, 1909, and organised by electing John H. Grant, chairman, and F. E. Fronczak, secretary. Several meetings were held during the year. A detailed memorandum statement of the board's work has been submitted by the chairman, at the several quarterly meetings of the society. During the present quarter the case of Mrs. Dr. M. E. Lane, has resulted in her indictment by the Grand Jury, and recent arraignment in Supreme Court, where she was held under $1,000 bail for trial in the January term of court.

On November 26, 1909, a warrant was sworn out for the arrest of one Ferdinand Moehlau, running a drug store at 9 Mohawk street, Buffalo, who it appears has been practising medicine by giving certain injection material, and performing urethral irrigation in a back room of his drug store on young men at $25.00 per case, one of the young men making affidavit upon which the arrest was made. The case was presented and tried in Police Court by our counsel; the defence put on the witness stand Dr. Monroe Manges, with offices in the Brisbane Building, who testified that he had filed prescriptions and a statement of urinalysis covering this particular case, with the druggist, on the latter's statement of the case, although he, Manges, had never seen the patient, and admitted that he received $15.00 from the druggist and had frequently done this in many such cases. As a result of this testimony Moehlau was discharged.

Generally summarising the following cases have been investigated by the board during the year—namely, F. J. Lambert, Atlantic or National Clinic or Dr. Merrow, Ebenezer Medicine Co.; Dr. Wm. F. Bell, Nature's Creation Remedy Co.; The Bessie Stokes case; C. Smith Paul, osteopathist; Mrs. Caroline Baily, alleged abortionist; Dr. De Courlander, Dr. Kelly Medical Co., The Vacuum Co., Dr. Porter Medical Co., Mrs. Dr. Mattie E. Lane, Mrs. Moritz, midwife; Mrs. Fischer, clairvoyant; Dr. osteopathist;[1] Professor William Hoffmann, Mechano-Therapist.

Total number of cases investigated, 17; total number of warrants sworn out, 8; total number arraigned in Police Court, 8; total number found guilty in Police Court, 1; total number held for action of the Grand Jury, 4; total number discharged in Police Court, 2; total number admitted to bail pending trial in Police Court, 1; number investigated by the Grand Jury, 4; number held for trial by Grand Jury, 2; number failed to indict by Grand Jury, 2; number arraigned in Supreme Court, 2; number found guilty and sentenced, Supreme Court, 1; number awaiting trial in Supreme Court, 1.

In addition to these cases, through the efforts of the board, a physician, alleged abortionist, in Niagara County, is now under trial at Lockport for suspected abortion on an Erie County woman; a fraud order issued by the postal authorities against a concern known as the Vacuum Co., and a hearing in the Dr. Ince case for reinstatement to medical practice, before a committee of the State Board of Medical Examiners, the opposition of your board resulting in postponement of the case to some future time,

1. So in copy.

to be determined if further facts in favor of said H. James T. Ince develop.

Regarding non-professional persons engaged in the practice of abortion, such as nurses and midwives, it is believed from experience gained that it is not within the province of the board to investigate and expend money in the prosecution of such cases, but all such complaints should be referred to the District Attorney of the County. It will be noticed that in the medical law only money expended by the society in the conviction of licensed practitioners can be reimbursed.

The council has, during the year, voted to the use of the chairman of the board the sum of seventy-five ($75.00) dollars, of which the chairman has expended sixty-one dollars and twenty cents ($61.20) covered by vouchers, and there is due, and not yet paid, one dollar and fifty cents ($1.50), making a total of sixty-two dollars and seventy cents ($62.70). A further expense incurred by Dr. George L. Brown, in investigating, as a member of the board, the Porter Medical Company, to the amount of ten dollars and seventy-five cents ($10.75) has been ordered paid, making a total expenditure of the year of seventy-three dollars and forty-five cents ($73.45).

The employment of Mr. Charles E. Doane, as counsel for the society, on the recommendation of the chairman was approved by the council in the early part of the year. Mr. Doane has rendered efficient legal services and, as has been the custom, an honorarium of one hundred dollars ($100.00), is recommended and the council has ordered the same be paid to him.

Respectfully submitted,

John H. Grant, Chairman.
Francis E. Fronczak,
Walter D. Greene,
George L. Brown.

The censors' report was adopted with recommendations contained therein, and the thanks of the society given the censors for their valuable services throughout the year.

The Committee on Legislation, through its chairman, Dr. F. Park Lewis, made a verbal report of work accomplished during the year and then presented resolutions as follows:

Whereas, Ophthalmia neonatorum, or birth infections of the eyes of infants, is rapidly destructive of sight when untreated, but is amenable to correct and persistent hospitable treatment; and

Whereas, The necessary treatment cannot be obtained in the homes of the poor, hence children are in danger of blindness from its neglect; and

Whereas, Aside from its humanitarian aspect, the cost to the commonwealth of educating and maintaining one blind individual amounts to many thousands of dollars; and

WHEREAS, The treatment is of brief duration and little cost; and

WHEREAS, No provision now exists whereby immediate and necessary treatment can always be promptly obtained for an infected infant

*Resolved,* That such cases are emergencies which admit of no delay.

*Resolved,* That the Medical Society of the County of Erie should take immediate steps to secure for the City Health Department authority to send the mother and child, when the eyes of the child are infected, to a hospital where proper attention can be secured, the expense to be borne by the city or by the county respectively, as the case shall be a city or county charge.

*Resolved,* That the Legislative Committee and the Council be directed to take such steps as may be necessary to have enacted a state law giving like authority to any health officer in whose jurisdiction such an infection may occur.

The following preamble and resolution were next presented:

WHEREAS, A large proportion of the births in the cities are cared for by midwives, many of whom are untrained, and whose services are unsanitary and inadequate; and

WHEREAS, There is no uniformity in the laws governing their practice in the state; therefore be it

*Resolved,* That the Medical Society of the County of Erie strongly favors the enactment of a uniform state law governing the practice of obstetrics by midwives and providing for their licensing, supervision, and legal control.

The report was accepted and the resolutions were adopted.

DR. HENRY R. HOPKINS, acting chairman of the Committee on Public Health, made the following report for the committee:

*To the Medical Society of the County of Erie:*

Your Committee on Public Health would report that the progress of preventive medicine during the year last past has been so distinctive and singular, that its review should excite the highest sense of satisfaction and pride in every member of our profession.

One of the wise men of the world, ex-president Eliot, in a recent discourse gave a forecast as to the intellectual problems of the nearby future. This forecast was exceedingly optimistic and hopeful; and high upon the roll of honor of the comfort-making and life-saving forces of the future, the speaker placed preventive medicine.

In his recent message to Congress, President Taft recognises the importance of preventive medicine, and emphatically urges the creation of a National Bureau of Health. This fact, alone, would be sufficient to make the year 1909 memorable.

However, we would urge upon the society the supreme importance of persistent and continued agitation of this question, more especially through our medical organisations,—county, state and national. It will ever be a cause for just pride and satisfaction that in this effort to nationalise the question of preventive medicine this society has borne a conspicuous part.

At home the more important work of the year has been the effort to increase the facilities of our city for the protection of the public health by means of additional hospital accommodations, more especially for those ill with communicable diseases,—and in this matter we are able to report progress which, if not in all respects equal to our most sanguine expectations, still is progress of quite material proportions. The plans which have matured and been persistently pushed during the year have included two proposed hospitals,—one a city hospital for the reception of patients even if such are afflicted with the more frequently acute communicable diseases, to be built in some central and generally accessible location in the city; and another, a hospital for the care of curable cases of tuberculosis, to be located outside the city, as directed by the requirements of the work.

The completion of these hospitals will add enormously to our power to protect the public from preventable disease and death, and that completion should be fostered by every effort of this society. The work of selecting a site for a hospital in the city is meeting the expected opposition, hence is progressing slowly. We feel certain that each member of the society will do his utmost to uphold the hands of your special committee having charge of this important work.

As you well know, our city is disgraced by the presence of an epidemic of scarlet fever of more than a year's duration, and also with an epidemic of measles of large proportions, unusual severity, and considerable mortality. The health office is struggling against this double menace to health and life as best it can, with an equipment absolutely lacking the means essential to success—namely, hospital accommodations.

The plans for a hospital for curable cases of consumption have progressed more rapidly. A site at Perrysburg in Cattaraugus County, has been selected and the money for its purchase—about $20,000—has been given the city by the chairman of the commission having the matter in hand, His Honor, James N. Adam, Mayor, and the purchase of the site so selected has been accomplished.

Your committee recommends that this society, through its proper officers, transmit to His Honor, Mayor Adam, our deep and wide, and high appreciation of the wisdom and unselfishness of this generous contribution to the "Life Saving Service" of our city.

Respectfully submitted,

Ernest Wende, Chairman.
H. R. Hopkins,
Edward Clark.

The report and recommendations it contained were unanimously adopted.

Dr. John D. Bonnar, chairman of the special committee on contagious diseases hospital, reported concerning the work of his committee, as follows:

*To the Medical Society of the County of Erie:*

The committee of fifteen appointed by your society to secure a municipal hospital for the care of such contagious diseases as diphtheria, scarlatina and measles, begs leave to report that through its efforts in conjunction with similar efforts upon the part of the cognate committee, appointed by the Academy of Medicine, secured the appropriation for such hospital in October, 1908, in the sum of $200,000.

In the early part of the summer of 1909, the Mayor's Commission chose the Best street site for such hospital at the price of $50,000, which, considering the fact that the lot contained seven acres of land in the very center of the district mostly benefited by such institution, its elevation, southerly exposure, and easy accessibility by parents, friends and physicians of the patients requiring such isolation and most suitable medical treatment, at once appealed to your committee which, in season, and often out of season, urged the acceptance of this site upon the Board of Aldermen, but thus far that body has not reached a vote upon the measure. We know you are all so familiar with the status of this case it is needless to recite its history in detail, hence we will merely draw your attention to the fact that the choice of site is still pending and will most likely devolve upon the incoming council and mayor to decide that matter.

With appreciation and compliments, with the zeal and ever-willing coöperation of the similar committee of the Academy of Medicine, we heartily pursue with them the object sought in securing for the hospital for contagious diseases, the most approved location which, at this time is the Best street site.

Your influence and presence when possible for acquiring this property for such hospital is solicited by your committee.

Respectfully submitted, on behalf of committee,

John D. Bonnar, Chairman.

The report was adopted, the thanks of the society extended to the committee for its labors, and the committee was continued.

Treasurer Lytle submitted his annual report as follows:

*To the Medical Society of the County of Erie:*

It is a pleasure to state that so much of the fiscal year as can be included in this report has been a very satisfactory one to the society financially. As the fiscal year closes with December 31, 1909, this report is incomplete to the extent of the business transacted at this meeting and which will be included in the completed report to be submitted to the auditing committee.

On May 28, 1908, order No. 40 for $3.00, was directed drawn payable to Dr. H. Y. Grant for return of fees. Notwithstanding repeated requests, Dr. Grant has not complied with the requirements ordered, and he has since resigned from the society. The treasurer recommends that the society direct order No. 40 cancelled and the funds returned to the treasury.

It is also a pleasure to announce that all members have paid their dues and assessments for 1909, hence no one will be dropped for non-payment of dues.

Respectfully submitted,

ALBERT T. LYTLE.

Dated, December 20, 1909.

### TREASURER'S REPORT—DECEMBER 20, 1909.

### Treasurer, Debtor.

| | |
|---|---|
| Balance on hand, December 31, 1909 | $ 366.97 |
| Unpaid, Order No. 40 | 3.00 |
| State Assessment for 1908 | 3.00 |
| State Assessment for 1909 | 1,158.00 |
| State Assessment for 1910 | 3.00 |
| County Dues for 1908 | 2.00 |
| County Dues for 1909 | 722.00 |
| County Dues for 1910 | 2.00 |
| Outing Fund, Special | 18.72 |
| Total | $2,328.69 |

### Treasurer, Creditor.

| | |
|---|---|
| Orders paid favor State Society | $1,110.87 |
| Orders paid for Secretary's Office | 167.87 |
| Orders paid for Treasurer's Office | 61.06 |
| Orders paid for Board of Censors | 88.75 |
| Orders paid for Membership Committee | 51.20 |
| Orders paid for Entertainment Committee | 166.55 |
| Orders paid for Legislative Committee | 18.00 |
| Orders Paid for Necrology Committee | 4.40 |
| Orders paid for sundry items | 179.00 |
| State Assessments, 1909—6 resigned | 18.00 |
| County Dues, 1909—6 resigned | 12.00 |
| Balance on hand | 451.86 |
| Total | $2,328.69 |

### Cash.

| | |
|---|---|
| Balance, December 31, 1908 | $ 366.97 |
| Receipts, 1909 | |
| State Assessments, 1908 | 3.00 |
| State Assessments, 1909 | 1,140.00 |
| State Assessments, 1910 | 3.00 |
| County Dues, 1908 | 2.00 |
| County Dues, 1909 | 760.00 |

| | | |
|---|---|---|
| County Dues, 1910 | | 2.00 |
| Outing Fund | | 18.72 |
| Unpaid Order No. 40 | | 3.00 |
| Total | | $2,298.69 |

**Disbursements, 1909.**

| | |
|---|---|
| Paid Orders Nos. 114 to 173 inclusive | $1,846.83 |
| Balance on hand December 20, 1909 | 451.86 |
| Total | $2,298.69 |

| | | |
|---|---|---|
| Members in good standing January 1, 1909 | | 311 |
| Member in good standing January 1, 1908, but dropped December 31, 1909, for non-payment of dues | 2 | |
| Members reinstated from those dropped December 31, 1909 | | 1 |
| Members reinstated from resignation, 1909 | 1 | |
| Members added new during 1909 | | 74 |
| Total | | 386 |
| Members died during 1909 | | 2 |
| Members resigned during 1909 | | 8 |
| Members in good standing December 20, 1909 | | 376 |
| Total | | 386 |

The report was received and filed and ordered referred to an auditing committee when appointed. On motion of Dr. Bennett, the thanks of the society were extended to Dr. A. T. Lytle for his excellent work. The recommendation of the treasurer to cancel a warrant in order to straighten out his books was adopted.

The president appointed Drs. Edward Clark and William Irving Thornton as a committee to audit the treasurer's accounts.

Dr. J. W. Grosvenor, chairman of the committee on necrology, presented the following memorials on the deaths of Dr. David C. Eisbein, Dr. John Dambach and Dr. William C. Krauss, and in doing so he expressed the gratification of the committee that among so large a membership, only three members of the society had died since the last annual meeting.

Report of the Committee on Necrology of the Medical Society of the County of Erie, for the year 1909.

*To the Medical Society of the County of Erie:*

Three members of this society have died since the last annual meeting—David C. Eisbein, John Dambach, and William C. Krauss.

### Obituary of David C. Eisbein, M. D.

The subject of this record was born in Germany, February 2, 1844. He died at his residence, 399 Broadway, Buffalo, N. Y., on December 24, 1908, at the age of 64 years, from hypertrophy of the heart with dropsy as a contributing cause of death. After graduating from a German gymnasium, he pursued the study of medicine in the medical colleges of Heidelberg, Vienna and Leipsic, receiving from each of these institutions a certificate for his study and work.

For his bravery in the German army during the Franco-Prussian war he received a cross of honor. After completing his medical studies in Germany, he came to this country, entered the University of Buffalo and was graduated from its medical department in 1868. Immediately after his graduation he went to the University of Louisville, Ky., as an assistant to Dr. Bodine and lecturer on medical subjects in that institution. Having filled these positions for one year, he returned to Buffalo where he practised his profession during the remainder of his life.

He was a medical examiner for some life insurance companies and, at one time, as district physician was connected with the Buffalo Board of Health.

He was a member of the Medical Society of the State of New York and of the Medical Society of the County of Erie, also a member of the Buffalo Sængerbund, the Orpheus, The Independent Order of Oddfellows, Ancient Order of United Workmen and Woodmen of the World.

Occasionally, Dr. Eisbein contributed articles to newspapers on medical subjects. Although a very busy man he found time to indulge in vacations and physical recreations. During his latter years he visited annually or biennially his native country and the old homestead, the last time in 1908. During these trips he visited Switzerland and engaged in Alpine climbing, a species of recreation of which he was very fond. On his last visit to that country in 1908 he climbed several mountains and the severe exertion affected his heart, perhaps thereby hastening his last sickness and death.

The funeral of Dr. Eisbein occurred at his residence December 28, 1908, the burial being at Buffalo Cemetery, Pine Hill. He is survived by his wife, Augustina, a son who is Dr. Arthur Eisbein, and five daughters, Mrs. F. L. Pfenning, Mrs. Adrienne Osborne Van Kraus, Christine, Adele and Clara Eisbein. From the preceding account it is not difficult to recognise that Dr. Eisbein was eminently qualified by education to enter upon the medical profession which he followed with assiduity and success.

### Obituary of John Dambach, M. D.

John Dambach, M. D., was born in Buffalo, September 6, 1843. His death occurred at the age of 66 years, September 15, 1909, at his residence, 417 Michigan street, Buffalo. The cause of his death was cerebral embolism.

He was of German extraction, his father, Justis Dambach, being a native of Germany and his mother, Anna Margaret Remlee, of Alsace, a French Province at the time of her birth. His early education was obtained in the public schools of Buffalo. He studied for the Lutheran ministry in Columbus, O., but never assumed the duties of a clergyman.

As a medical student he studied in the office of the late Dr. C. C. F. Gay, of Buffalo. Having completed his medical studies in the Medical Department of the University of Buffalo before his age permitted him to be graduated, he did not receive his diploma from that institution until the year 1868. Soon after the completion of his medical course he acted as an assistant surgeon in the civil war and continued in that capacity till the close of the war in 1865.

Immediately after this service he took up his residence in Trinity, La., and there practised his profession till 1872. During this period he was married to Virginia A. Tiffee. Returning to Buffalo in 1872, he continued his professional career in this city until his death. His medical practice was large and covered a peroid of more than forty years.

For many years he was a member of the Medical Society of the County of Erie, and of the Medical Society of the State of New York. He was also a member of the fraternal societies, Knights of Honor, Foresters and Ancient Order of United Workmen, of the last named a medical examiner. At one time Dr. Dambach was a member of A. J. Meyer Post No. 139, Department of New York, Grand Army of the Republic and its Commander for four official terms beginning with 1894.

The funeral services were held at his residence, September 19, 1909, the officiating clergyman being Rev. Dr. F. A. Kahler. The delegates appointed to represent the Medical Society of the County of Erie were Drs. H. R. Hopkins, A. H. Briggs, Edward Clark, J. W. Grosvenor, John H. Grant. There was also in attendance at the funeral a large representation of veterans of the civil war besides friends and relatives of the deceased. He is survived by a widow, Virginia A. Dambach, two daughters, Mrs. Pliny B. McNaughton and Mrs. J. Albert Beirlein. The druggist, W. C. Dambach was his brother.

By his professional confreres, who knew him well, Dr. Dambach was held in high esteem for his professional attainments, his conscientious spirit and loyalty to the medical profession.

He had a cheerful and generous disposition; cared but little for the gaieties of social life; took a deep interest in his home, its comforts and society; his leisure hours were spent mostly at his fireside; he was a *home* man.

### Obituary of William Christopher Krauss, M. D.

William Christopher Krauss, was born in Attica, N. Y., October 15, 1863. His father, Andrew G. Krauss and his mother, Magdalene (Foote) Krauss were natives of Germany. In early life Dr. Krauss suffered from heart trouble. Several years previous to his death his cardiac difficulty assumed a definite form and became so severe that on July 9, 1909, he sailed from New York for Europe for the purpose of consulting eminent physicians. Returning to this country, after a stormy ocean voyage, he arrived in New York city on September 20, 1909, and died there on the following day, September 21, 1909, from endocarditis, at the age of 45 years.

The following record appears in the Cornell University Register of Holmes and Williams, published in 1905. "Krauss, William C., physician; specialist in nervous and mental diseases, 479 Delaware Ave., Buffalo, N. Y., b. Attica, N. Y., October 15, 1863. Prep. Attica Union School (valedictorian) '80. Cornell (course of medical preparatory and science and letters) B. S. '84; received credit for six years work at Cornell; honor class Bellevue Hosp. Med. Coll. '86; Magna cum laude Univ. Berlin, Germany, with degree M. D., '88. Horace K. White prize in veterinary science (Cornell) '83; special final honors '84; publication of thesis by faculty on "Psyche;" work done on insect's brain in Professor Comstock's laboratory attracted special attention; elected to Sigma XL '03; member Class Day Committee; Associate editor BUFFALO MEDICAL JOURNAL; also of *Neurologisches Centralblatt,* Berlin, Germany, and of *Journal of Nervous and Mental Diseases,* 1890-1900; author of one hundred papers on medical subjects, especially relating to the nervous system. First to study effects of high voltage upon the brain; designer of neurotopographical bust; asthesiometer; pedodynamometer, and other instruments for use in study of nervous diseases. Medical Superintendent Providence Retreat for Insane; appointed by Governor Odell, a member state board of visitors to Buffalo State Hosp., 1900; organiser and secretary Buffalo Acad. Med., 1891-94; secretary Buffalo Obstetrical Soc., 1890; president Buffalo Microscopical Club, 1892; secretary Amer. Micros. Soc., 1896-99; president Amer. Micros. Soc., 1899; president Med. Association Central N. Y., 1898-1899; president Erie County Med. Soc., 1903; neurologist to the following Buffalo hospitals: Buffalo General, Erie County, Emergency, German, German Deaconess, Woman's, Buffalo Eye and Ear Infirmary, also to Batavia (N. Y.) Hosp.; Brooks Memorial Hosp., Dunkirk, N. Y. Professor of Pathlogy Niagara Univ., Buffalo, 1890-94; professor of nervous diseases, 1894-99; member Buffalo Club; Univ. Club; Med. Club; Buffalo Alumni Association; Med. Union; Buffalo Hist. Soc.; Fellow Royal Microscopical Soc., London; member Amer. Micros. Soc.; Amer. Neurological Association; Amer. Public Health Association; N. Y. State Med. Soc.; Med. Association Central N. Y.; Erie County Med. Soc.; Buffalo Acad. Med.; Buffalo Soc. Natural Sciences; honorary member Onondaga, Chautauqua, Cattaraugus, Niagara and Wyoming County medical societies.

Married September 4, 1900, at Salamanca, N. Y., Clara Krieger; children, Magdalene, Alma and William A."

The above extract is a concise record of Dr. Krauss's professional and social life. He also pursued his medical studies at Munich and Paris. At one time he was a lecturer at Cornell University. After completing his medical studies abroad, he began his professional career in Buffalo, and continued it here during the remainder of his life.

From this record it is not difficult to conclude that he was a tireless worker. His specialty was neurology. In his investigations it was his controlling purpose to arrive at the truth. He marshaled his facts in regular order and gave to each one its proper influence in producing the final result; he eliminated the chaff from the wheat.

He was a prolific writer, as shown by being an associate editor of several medical journals, the author of more than one hundred papers on medical subjects, his translation from the German of Professor Mendel's Textbook of Psychiatry, authorship of a work on Tumors of the Spinal Cord completed just before his death, but unpublished. His literary style was clear and forcible, his deductions fortified by arguments strong and convincing.

His services as a consultant in nervous diseases were frequently employed and highly valued. He was a medical examiner for several insurance companies, Germania, New York Life, and Berkshire. As an expert witness in cases of insanity and accidents resulting in injury to the nervous system his testimony was lucid and positive and received marked attention. The many medical societies of which he was a member and of some of which he was the president and leading spirit, had his uniform attendance and hearty support. Notwithstanding his very busy professional life, he found time to enjoy social amenities and recreations and did his share in rendering successful the fraternal societies to which he belonged.

The funeral services were held at his residence, 479 Delaware Avenue, on September 23, 1909. The officiating clergyman was the Rev. Dr. Charles Earle Locke, a long time friend, who bore earnest testimony to the fact that Dr. Krauss was *"a man, a man,"* whose character was invincible for the right. The following members of the Medical Society of Erie County were appointed as delegates to attend the funeral: Doctors Himmelsbach, Cohen, F. P. Lewis, Gram, Clark, Lothrop, Starr, Bennett, Putnam, Frederick, Matzinger, C. S. Jewett, Bowerman, E. J. Meyer, J. H. Potter, Hurd, Hartwig, Hayd, W. D. Greene, Grosvenor. The burial was at Attica, New York, under the auspices of Washington Lodge, No. 240, F. and A. M.

In 1890, Dr. Krauss was married to Miss Clara Krieger by whom he is survived and by three children, Magdalene, Alma and William A.

Dr. Krauss took a high rank as a medical practitioner. His examinations of cases were minute and exhaustive. He constantly aimed to develop in himself the highest and best of which he was capable. In his intercourse with his medical brethren, he was courteous, considerate and helpful.

Dr. William Christopher Krauss will long be held in memory for the uprightness of his character, large attainments and loyalty to the medical profession.

The society may appropriately be congratulated on the low death-rate of its membership since its last annual meeting. The Committee on Necrology notes with satisfaction that a suitable register has been purchased for the obituary records of the society.

J. W. GROSVENOR,
WM. T. GETMAN.

A vote of thanks was tendered to Dr. Grosvenor for his excellent work.

The election of officers was then proceeded with, the chair having appointed H. G. Hopkins and L. M. Francis as tellers. Nominations being called for, Grover W. Wende was nominated for president; and by vote of the society, the secretary was directed to cast the ballot of the society for his election. The secretary having done so, the chair declared Grover W. Wende duly elected president for the year 1910.

Dr. Bernard Cohen was nominated for first vice-president, and the secretary was directed to cast the ballot of the society for his election; whereupon, he was declared duly elected.

Dr. Edward Clark nominated John D. Bonnar for second vice-president. Dr. Bennett stated that the towns had not been represented for some time, and therefore nominated Frank Helwig, of Akron, as second vice-president. Dr. Bonnar then withdrew in favor of Dr. Helwig and moved that his election be made unanimous, whereupon the secretary was directed to cast the vote of the society for the election of Dr. Frank Helwig as second vice-president, and Dr. Helwig was declared duly elected.

By vote of the society, the president was directed to cast the ballot for the election of Dr. F. C. Gram as secretary, whereupon he was declared duly elected.

The secretary was then directed to cast the ballot of the society for Albert T. Lytle as treasurer, and the vote being cast, Dr. Lytle was declared duly elected.

The secretary was directed to cast the ballot of the society for the same present incumbents as Censors, as follows; Drs. John H. Grant, DeLancey Rochester, Walter D. Greene, Francis E. Fronczak and George L. Brown, whereupon they were declared duly elected.

The secretary was directed to cast the ballot of the society for Dr. F. Park Lewis as Chairman of the Committee on Legislation.

The secretary was directed to cast the ballot of the society for Dr. Ernest Wende as Chairman of the Committee on Public Health.

The secretary was directed to cast the ballot of the society for Dr. Thomas H. McKee as Chairman of the Committee on Membership.

Nominations were then called for four delegates to be elected to represent this society in the State Society during the year 1910-11. Nominations were made as follows: Drs. H. R. Hopkins, B. Cohen, G. W. Wende, G. L. Brown, M. Hartwig, William H. Thornton, S. A. Dunham, Marshall Clinton, Julius Ullman, F. E. Fronczak, J. D. MacPherson, W. M. Ward and A. W. Hurd.

Nominations were declared closed. Drs. Hopkins and Ullman withdrew their nominations, stating that they would be unable to serve if elected. The tellers were directed to prepare ballots, and when the votes were counted, the following four members were declared elected as delegates to the state society for the years 1910-11: Drs. G. W. Wende, A. W. Hurd, William H. Thornton, and B. Cohen.

President Wall read the following amendment to the rules as presented by the council and approved of by the state society, which was then unanimously adopted:

Chapter XIII, Section 2.—Rules and regulations for the government of the society and the administration of its affairs, not repugnant to the constitution and by-laws of the Medical Society of the State of New York, and any of these by-laws may be adopted, changed, amended, or suspended at any regular meeting by a three-fourths vote, twenty members being present.

Dr. McKee's recommendation as to a permanent place of meeting was taken from the table and referred back to the committee for further investigation and report. A resolution was adopted empowering the council to elect an alternate whenever a delegate to the state society is unable to attend a meeting, and this resolution was made a standing rule. On motion of Dr. Grosvenor, an honorarium of $50.00 was presented to the secretary and a like amount to the treasurer in acknowledgment of their services during the past year.

The retiring president, Dr. Charles A. Wall, called the newly elected president to the chair and delivered his annual address. On motion of Dr. Bonnar, the thanks of the society were extended to the retiring president, and he was asked to publish his paper so that its historic value may be of further benefit to the members.

The retiring president then relinquished his office to president-elect Dr. Grover W. Wende who, on assuming the duties, briefly thanked the society for the compliment bestowed upon him. As the hour was late, and a collation was still to be served, the scientific part of the program was, on motion, deferred to the next meeting. Adjournment then followed.

